# Photonic Band Gap Engineering by Varying the Inverse Opal Wall Thickness

**DOI:** 10.3390/ijms252312996

**Published:** 2024-12-03

**Authors:** Dániel Attila Karajz, Levente Halápi, Tomasz Stefaniuk, Bence Parditka, Zoltán Erdélyi, Klára Hernádi, Csaba Cserháti, Imre Miklós Szilágyi

**Affiliations:** 1 Department of Inorganic and Analytical Chemistry, Faculty of Chemical Technology and Biotechnology, Budapest University of Technology and Economics, Műegyetem rkp. 3, H-1111 Budapest, Hungary; karajz412@edu.bme.hu (D.A.K.);; 2 Faculty of Physics, University of Warsaw, 5 Pasteura St., 02-093 Warsaw, Poland; tomasz.stefaniuk@fuw.edu.pl; 3 Department of Solid State Physics, Faculty of Sciences and Technology, University of Debrecen, P.O. Box 400, H-4002 Debrecen, Hungaryzoltan.erdelyi@science.unideb.hu (Z.E.); cserhati.csaba@science.unideb.hu (C.C.); 4 Institute of Physical Metallurgy, Metal Forming and Nanotechnology, University of Miskolc, H-3515 Miskolc, Hungary; klara.hernadi@uni-miskolc.hu

**Keywords:** atomic layer deposition, inverse opal, photocatalysis, photonic crystal, carbon nanosphere

## Abstract

We demonstrate the band gap programming of inverse opals by fabrication of different wall thickness by atomic layer deposition (ALD). The opal templates were synthesized using polystyrene and carbon nanospheres by the vertical deposition method. The structure and properties of the TiO_2_ inverse opal samples were investigated using Scanning Electron Microscope (SEM) and Focused Ion Beam Scanning Electron Microscopy (FIB-SEM), Energy Dispersive X-ray analysis (EDX), X-ray Diffraction (XRD) and Finite Difference Time Domain (FDTD) simulations. The photonic properties can be well detected by UV-Vis reflectance spectroscopy, while diffuse reflectance spectroscopy appears to be less sensitive. The samples showed visible light photocatalytic properties using Raman microscopy and UV-Visible spectrophotometry, and a newly developed digital photography-based detection method to track dye degradation. In our work, we stretch the boundaries of a working inverse opal to make it commercially more available while avoiding fully filling and using cheaper, but lower-quality, carbon nanosphere sacrificial templates.

## 1. Introduction

Today’s challenges in science include the research of technologies allowing environmentally-friendly purification of waters and surfaces. Photocatalytic materials using the energy provided by the sun can be a solution to this problem and they have been researched since the experiment of Fujishima and Honda in 1972 [1]. They achieved water splitting with photoelectrodes made of TiO_2_, which is the most-researched photocatalyst. TiO_2_ is an excellent material, but it has its drawbacks, most importantly its band gap energy of 3.2 eV, which is in the UV region of the light spectrum. Due to the high stability and reactivity of TiO_2_ [2], it can be used for photocatalysis by artificial UV irradiation [3], but it is less effective when using natural sunlight. A variety of strategies are studied to overcome the high energy of the band gap: doping TiO_2_, hence decreasing the band gap [4]; applying TiO_2_-based composites; combining TiO_2_ with a lower band gap energy material, which can include metals and carbon nanomaterials, forming a heterojunction [5]; dye sensitization of TiO_2_, i.e., using various organic dyes as mediators, which can be excited with visible light acting similarly of heterojunctions [6]; and finally, the nanostructure of the TiO_2_ can be designed to help photocatalysis, having larger specific surface area or special optical properties [7]. Our current work focuses on the last-mentioned strategy.

Photonic materials, especially inverse opal crystals of TiO_2,_ are frequently studied in photocatalysis for multiple reasons. The bottom-up manufacturing of these materials contains similar steps: production of the opal crystal (sacrificial template), infiltration with photocatalytic material, removing the template (usually via calcination or dissolution). The most commonly used building blocks for opal crystals are SiO_2_ or polymer (PSA, PMMA) nanospheres [8]. Natural colloidal self-assembly can form opal crystals, but this method is too slow for practical use. Other faster, but still cheap methods are e.g., enhanced gravitational sedimentation with other forces like filtration, vertical deposition, centrifugation, slit filling and pressing [9]. The second important step is the infiltration of the opal crystal, which can be done by the sol-gel method [10], chemical bath deposition (CBD) [11], chemical vapor deposition (CVD) [12] and atomic layer deposition (ALD) [13].

During the production of the inverse opal photonic crystal, there are multiple parameters that can be used to modify its structure; hence the photonic band gap. The two most powerful tools to achieve the desired band gap are varying the size of the nanospheres and the choice of the infiltration material. The diameter of the nanospheres directly correlate to the pore size of the inverse opal crystal and effects the air void-to-bulk material ratio, namely the filling fraction. The position of the void spheres inside the bulk structure in relation to each other, the lattice constant, also affects the optical properties. On the other hand, the material property of the crystal also changes the band gap due to its dependence on the refractive index of the solid, bulk phase and the void spheres [14,15]. Multicompound inverse opals can be also used to achieve different photonic band gap structures and increase the photocatalytic efficiency, e.g., TiO_2_ can be combined with ZnO [13,16].

According to the literature, the primary strategy to enhance photocatalysis with photonic crystals, such as inverse opal, is to overlap the red edge “slow” photons (localized in the semiconductor) with the semiconductor’s band gap to increase the electron-hole generation [17]. On the other hand, coinciding the blue edge “slow” photons (localized in the void spheres) with the semiconductor’s band gap also works to great effect [18]. In connection to this article positioning the “slow” photons in the visible region is also a viable path [19,20]. Additionally, Harakeh M. E. et al. showed that introducing disorder by using multiple different-sized nanospheres for the opal template can increase the visible light photocatalytic efficiency [21].

In the context of our work, the important parameter is the filling fraction of the inverse opal, which affects the photonic band gap position. The reason is that it has a direct connection with the refractive index:

(1) $$n_{\mathrm{avg}}=\Phi n_{\mathrm{sphere}}-\left(1-\Phi \right)n_{\mathrm{background}},$$

where *n*_avg_ stands for the average refractive index of the inverse opal and the *Φ* solid fraction. Notably, this generalized equation can be used for opals (sphere–nanosphere, background–interstitial space) and inverse opals (sphere–void sphere, background–solid walls). The relation between the average refractive index, hence the filling fraction relation, to the photonic band gap position is the following in a simple case (first order reflection, 90° angle irradiation on the [1 1 1] crystal plane:

(2) $$\lambda =1.633D\sqrt{n_{\mathrm{avg}}^{2}-1},$$

where *λ* is the position of the photonic band gap and *D* is the pore spacing [15,22]. Changing the filling fraction of an inverse opal influences the position of its photonic band gap. X. Yu and co-workers made changes to the filling fraction of metal inverse opal using post-preparation etching, which decreases the wall thickness and shifts the photonic band gap [23].

Other strategies are also investigated in the modification of the band gap structure of inverse opals, e.g., using noble metal nanoparticles. Gold, silver and platinum nanoparticles can change the band gap structure due to their high refractive index and increase the photocatalytic property [24,25,26,27]. The photonic characteristics can be changed via doping the building material of the inverse opal with various elements or ions, e.g., TiO_2_ inverse opals were doped with N, F elements and Tb^3+^, Yb^3+^ and Er^3+^ metal ions [28,29,30,31,32]. Another successful method is the sensitization of the photonic crystal with quantum dots made of semiconductive materials, and this way further fine-tuning of the photonic band gap is possible. Cadmium chalcogenide quantum dots like CdS and CdSe were used in inverse opal materials [33,34,35].

Another method to change the photonic band gap structure is the use of multiple, different-sized nanospheres to build the opal crystal, namely using binary colloid crystals as templates. Using a secondary nanosphere can make additional holes in the structure, and this shifts the photonic band gap with the ratio of the large and small spheres; in addition, it can increase the photocatalytic performance due to higher specific surface area [36,37,38].

Our work aims to explore a further possible way to alter the photonic band gap: with the partial, programmed infiltration of the colloid crystal opal template, which can be achieved with atomic layer deposition. Usually, the goal is the complete infiltration of the opal crystal, which is possible until the smallest interstitial site (trigonal) is filled; this also means that there will be left over holes. From the used nanosphere’s diameter, it is possible to calculate, how thick a layer needed to be deposited to fill the template, by closing the trigonal interstitial sites [39] and adjusting the layer’s thickness from the GPC (growth per cycle) [40]. Complete infiltration can be indicated by the closing of the surface porosity of the opal [41]. During the infiltration of an opal crystal, the deposition happens between the nanospheres up until the smallest holes (trigonal interstitial spaces) are closed. After this, only the outer surface of the opal is coated, and this causes an excess film deposition on the top surface of the inverse opal sample with a microbalance; this can be detected [42,43]. We expect that in the case of insufficient filling of the opal crystal voids, there will be leftover remnant holes, that can affect the photonic band gap, somewhat similar to using binary colloids. Accordingly, in this study the opal colloid crystals were made with the vertical deposition technique from polystyrene and carbon nanospheres, then three TiO_2_ inverse opals with different layer thicknesses (16.5 nm, 34.3 nm and 49.7 nm) were obtained by adjusting the ALD cycle numbers. The samples were characterized by SEM-EDX, XRD and UV-Vis spectrometry, as well as by Finite Difference Time Domain (FDTD) simulations. Surface photocatalytic activity was tested by Raman spectroscopy methods used in earlier research [20], and a newly developed photographic method was tested as well.

## 2. Results

**Figure 1:**
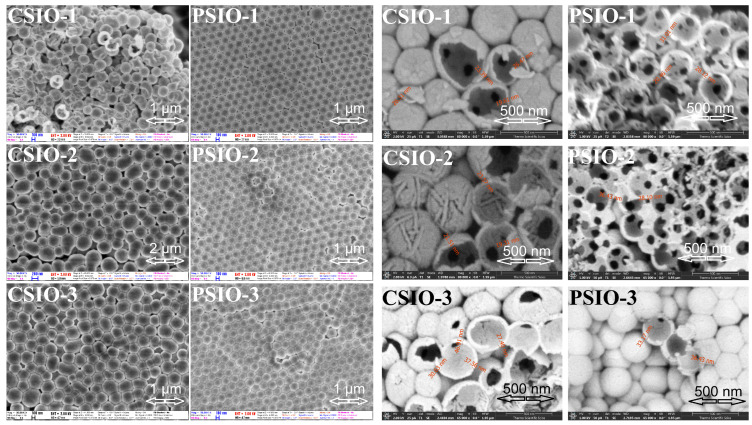
SEM images (**left**) and FIB-SEM images (**right**) of TiO_2_ inverse opal samples. CSIO–inverse opal made using carbon nanospheres opal as template PSIO–inverse opal made using polystyrene nanospheres opal as template 1, 2 and 3–16.5 nm, 34.3 nm and 49.7 nm deposited TiO_2_ layer by ALD.

SEM pictures of TiO_2_ inverse opals made using carbon (Figure 1 images of CSIO) and polystyrene (Figure 1 images of PSIO) nanospheres are presented below. A higher order of structure is achievable using a polystyrene template compared to carbon one which is due to the smaller deviance in the diameter of the polystyrene nanospheres. The opal shows a hexagonal structure on the surface, which indicates a face-centered cubic arrangement of the nanospheres. The FIB-SEM images reveal the inner, hollow structure of the inverse opals.

The results of the EDX measurements (Table 1) show that the main components of the samples are oxygen, silicon and titanium, and secondary components such as Na, Mg, Al and Ca were also detected. The high titanium presence indicates that the titanium dioxide deposition was successful; the significant silicon content originates from the microscope glass, and the same can be assumed of the Na, Mg, Ca and Al. The reason for this is that usually, the EDX penetration depth is 0.5–1 µm, and despite the inverse opal’s thickness being around 5 µm, it is highly porous. The absence of carbon and chloride indicates that the removal of the templates was efficient and that residual TiCl_4_ ALD precursor was not detectable.

**Figure 2:**
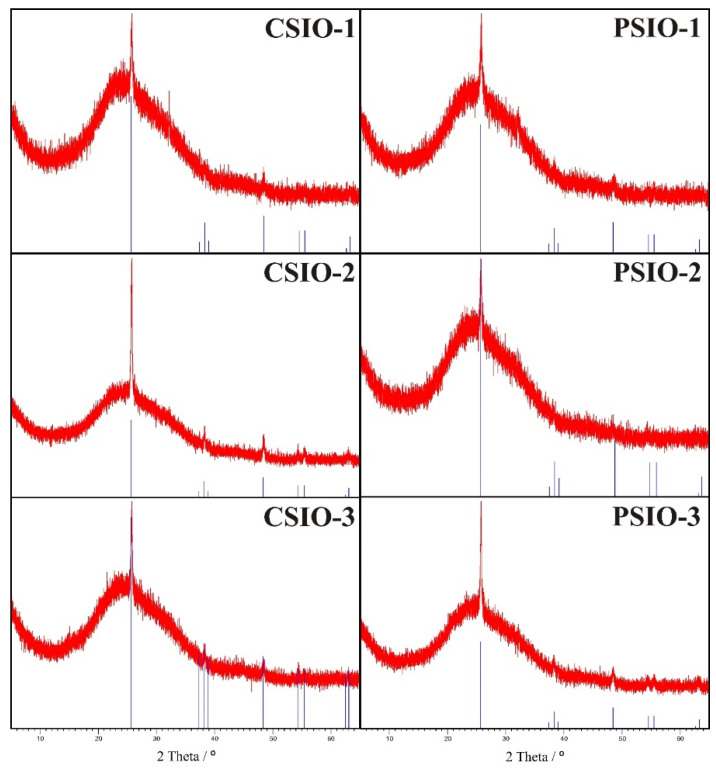
XRD diffractograms of the TiO_2_ inverse opal samples.

The XRD analysis (Figure 2) of the samples shows that the ALD-deposited TiO_2_ is crystalline and is in the anatase form with peaks at 25.7°, 37–39°, 48.5°, around 55°, 62.5° and 63.5° all indicating this (ICDD: 98-003-1064). The amorphous background is due to the microscope glass substrate.

**Figure 3:**
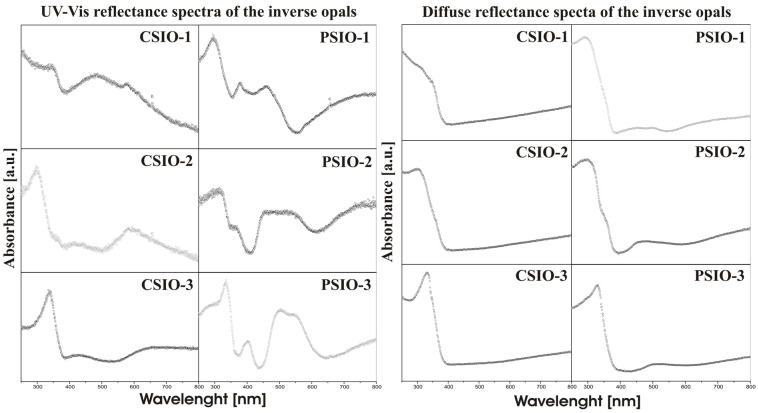
UV-Vis absorbance spectra of the TiO_2_ inverse opal samples in reflectance and diffuse reflectance modes.

In Figure 3, we present the measured directional reflectance spectra (left) and diffuse reflectance spectra (right) of the studied samples in the UV-Vis range. At first glance, the absorption edge of TiO₂ is observable in both types of measurements. However, there is a significant qualitative difference between the spectra for longer wavelengths. The directional reflectance measurements, performed using an optical fiber spectrophotometer, reveal a complex structure. The photonic bandgap is visible for all samples, with absorption edges located as follows: CSIO-1 to CSIO-3 at 390 nm, and PSIO-1 to PSIO-3 at 420 nm, 410 nm, and 430 nm, respectively. A potential “slow” photon-induced absorption increase is also noticeable. In some samples, more than two peaks are observed: for CSIO-1, peaks occur at 480 nm and 580 nm; for CSIO-2, around 420 nm and 580 nm; and for CSIO-3, at 425 nm and 650 nm. Similarly, for PSIO-1, peaks are observed at 380 nm and 465 nm; for PSIO-2, a peak merges with the absorption edge at 360 nm, and a broad peak (possibly two overlapping peaks) appears between 460 nm and 540 nm. For PSIO-3, a peak is observed at 400 nm, with merging peaks at 505 nm and 550 nm. In contrast, the diffuse reflectance spectra, obtained using an integrating sphere, show much less pronounced photonic features in the PSIO samples, or they disappear entirely, in the case of the CSIO samples. This observation confirms that the features seen in the directional reflectance measurements are associated with the structural characteristics of the samples rather than the intrinsic properties of the material itself.

**Figure 4:**
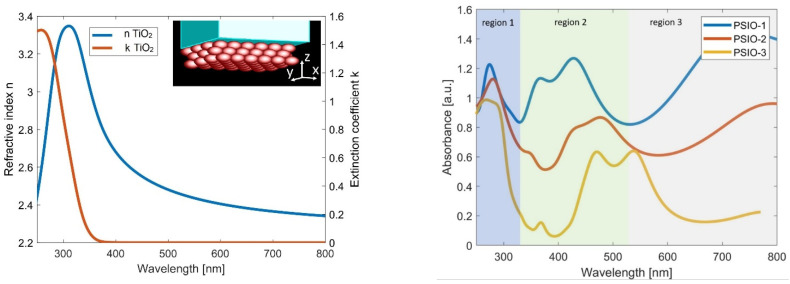
(**Left**): The refractive index and extinction coefficient of thin TiO_2_ layer, deposited using ALD process [44]. The inset presents the schematic of the opal structure, used in numerical simulations. (**Right**): Absorbance of PSIO samples calculated using FDTD method. The thicknesses of TiO_2_ layers, in particular, inverse opal geometries, correspond to the ones measured in the experiment: 16 nm (blue curve), 34 nm (red curve), 49 nm (orange curve). The shaded parts of the graph correspond to three different types of the optical response of the structure (marked for PSIO-1 sample).

**Figure 5:**
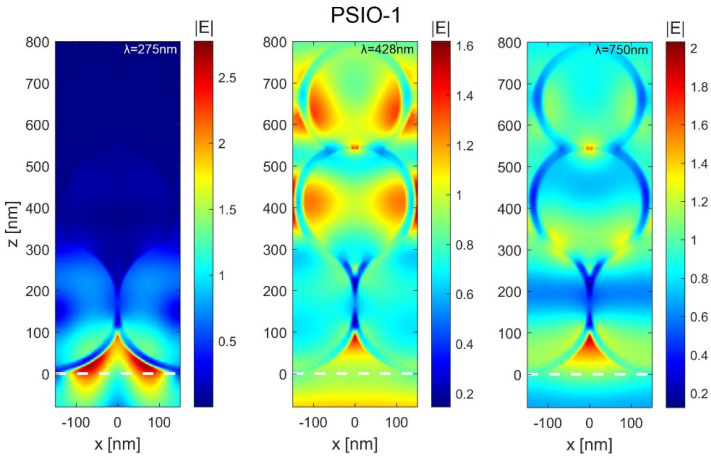
The electric field distributions calculated for a single cell of PSIO−1 sample for *λ* = 275 nm (**left**), *λ* = 428 nm (**middle**) and *λ* = 750 nm (**right**) wavelength. The colormaps are normalized to the amplitude of the illuminating plane-wave. The white line indicates the plane tangent to the first layer of nanospheres.

To understand the measured optical response of the inverse opals, we have performed Finite Difference Time Domain (FDTD) simulations. The general schematic of the structure under study is presented in the inset in Figure 4 (left). The TiO_2_ shells are tightly packed in three layers in FCC (111) lattice. We assumed that the geometry and dimensions of the inverse opal structure and the thickness of TiO_2_ layers are similar to those described and measured in the experimental section (see Section 4 for more details). In Figure 4 (right), we show the absorbance spectra calculated for the polystyrene nanosphere-based inverse opal (PSIO). The obtained curves are in good qualitative agreement with the measured reflectance spectra presented in Figure 3 (second column). Judging from the observable trends, we can distinguish three regions (marked accurately for the PSIO-1 sample by shaded areas in Figure 4 (right) in which different mechanisms govern the optical response of the inverse opals. In the first region (blue-shaded), the well-defined maximum in absorbance occurs due to the absorption of TiO_2_ material itself (see Figure 4 left). The position of the maximum does not change significantly between inverse opals with different geometrical parameters. By analyzing the electric field distributions for λ = 275 nm wavelength (Figure 5 left), it can be noticed that the illuminating wave does not penetrate the structure, and most of the light is absorbed in the first layer of the spheres. The absorbance curves in the second region are far more complex. Depending on the thickness of the TiO_2_ layer, there is a different number of peaks in the spectra. Moreover, with the increasing width of the TiO_2_ shell, the absorption region shifts towards the longer wavelengths. The study of the electric field distributions for the λ = 428 nm wavelength (Figure 5 middle) reveals a large number of resonances in the volume of the inverse opal structure. On these grounds, it can be said that in the second region, the optical response of the structure highly depends on the geometrical parameters. The initial size of the polystyrene spheres and the thickness of the TiO_2_ nanofilm determines the position, number and strength of the absorption peaks. Finally, we observe only a single absorption maximum in the third region for all three investigated samples. This time, the electric field distributions calculated for λ = 750 nm wavelength (Figure 5 right) show that the inverse opal structure starts to behave as a single slab with effective optical properties. It is also worth mentioning that in all three regimes, a light-intensity hot spot is generated in the cavity between the spheres in the first layer of the inverse opal.

**Figure 6:**
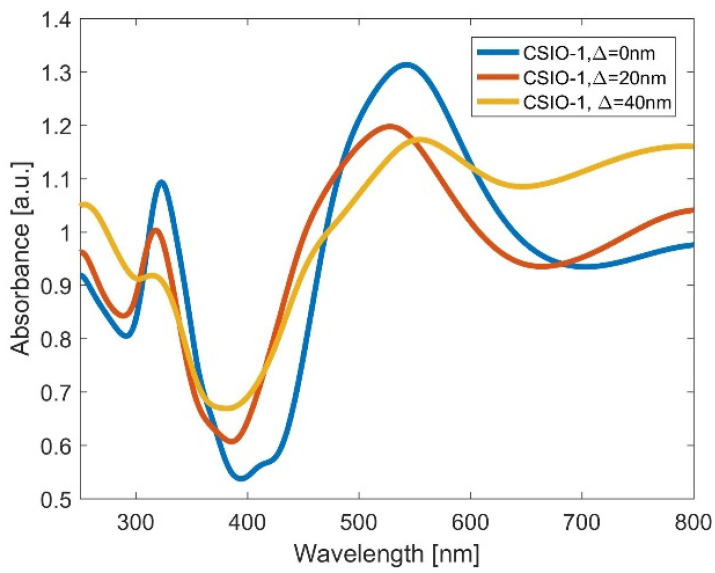
Absorbance of CSIO-1 sample calculated using FDTD method. Different curves correspond to samples with different degrees of disorder: (blue) no disarrangement, (red) Δ = 20 nm, (yellow) Δ = 40 nm.

In the course of the numerical analysis, it became apparent that in the case of CSIO samples, it is much more difficult to retrieve the same features than the ones observed in measured spectra (first column of Figure 3). We believe the problems are associated with the cells’ poor arrangement and size dispersion in carbon nanosphere-based inverse opal. To verify this hypothesis, we ran several simulations in which we studied the influence of disorder on the absorbance spectrum. In Figure 6, we present the results for the CSIO-1 sample. From SEM images, we assumed that the average sphere size is around 400 nm and the TiO_2_ layer thickness is equal to 16 nm. The introduced Δ parameter corresponds to the maximum variation in size of each nanosphere and deviation in its spatial position with respect to the regular lattice. The simulations proved that the introduction of disorder significantly modifies the optical spectrum. In particular, the geometrically dependent features from region 2 merge with those in region 3 and become indistinguishable.

**Figure 7:**
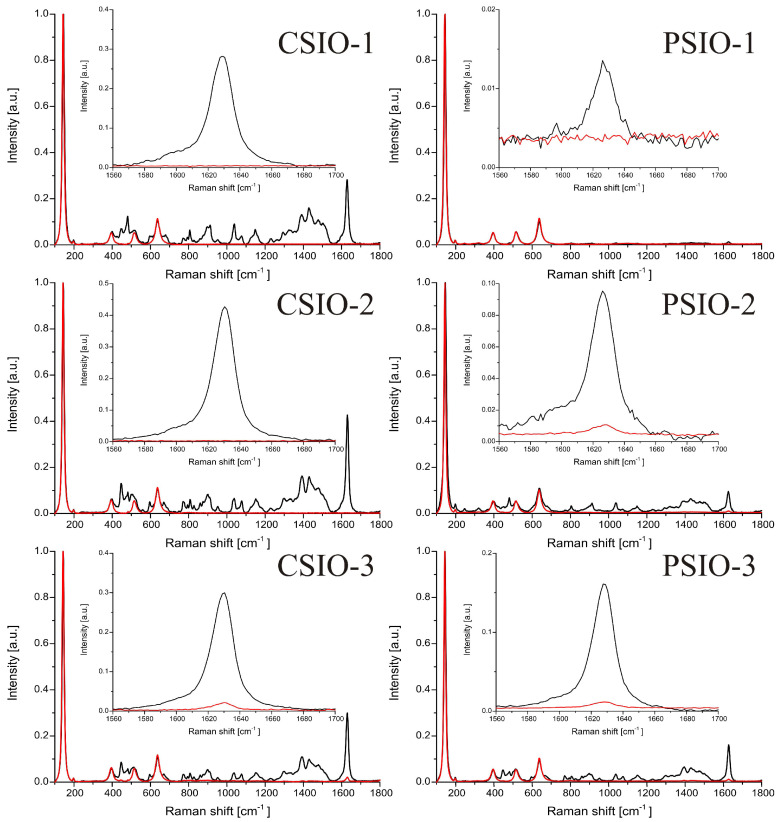
Raman spectra of the methylene blue dried on the surface of the samples before and after the 19-h irradiation with the highlight of the most intensive methylene blue peak.

The photocatalytic experiments followed by Raman spectroscopy (Figure 7) shows that after the 19 h UV irradiation, the most intensive methylene blue peak’s intensity significantly dropped or even almost fully flattened. The methylene blue was dropped, then dried on the surface of the sample during the experiment. In the case of CSIO-1, CSIO-2 and PSIO-1, the methylene blue peak is hardly observable after the 19 h irradiation; on the other hand, the CSIO-3, PSIO-2 and PSIO-3 samples had a noticeable flat peak. The photocatalytic efficiency was calculated by comparing the area of the peaks before and after the experiment. The results (Table 2) show that for all samples, at least 94% of the methylene blue degraded and, in the case of CSIO-1 and CSIO-2, even less methylene blue remained at the surface of the sample. The PSIO-1 showed a comparable remnant of the model solvent without a clearly visible peak; this might be due to the low intensity of the peak, where the measurement noise became significant.

**Figure 8:**
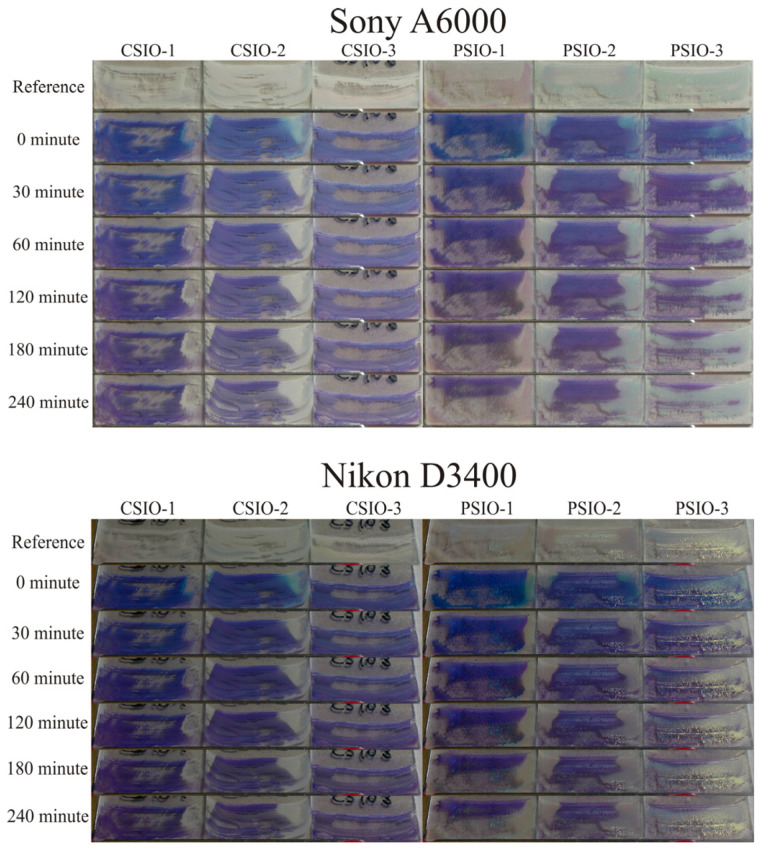
Images taken by the Sony A6000 (**top**) and Nikon D3400 (**bottom**) cameras during the photocatalysis experiment.

**Figure 9:**
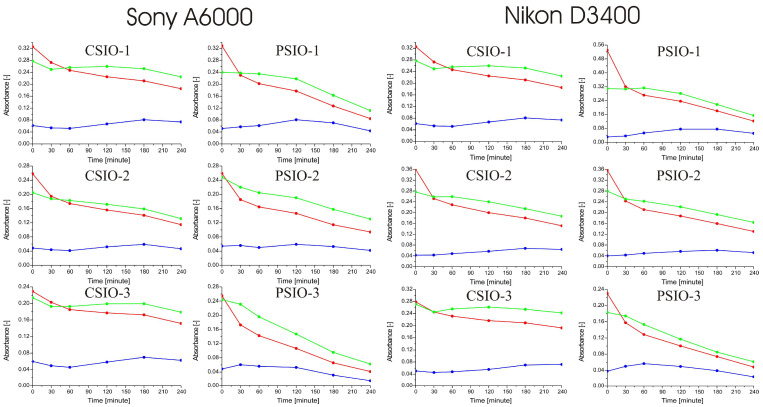
Summary of the photocatalysis tests using Sony A6000 (**left**) and Nikon D3400 (**right**) cameras. The spectrums show how the absorbance of red, green and blue color of the pixels changes over the 4 h long experiment.

Photocatalytic studies of the samples were done by using a Sony A6000, which is an entry-level digital single-lens reflex (DSLR) camera and a Nikon D3400, which is an entry-level mirrorless camera. The first row of images (Figure 8) shows the samples before dropping the methylene blue solution on their surfaces and drying it. The photocatalytic degradation of the methylene blue is observable with the rest of the images; in most cases, the degradation of methylene blue is visible in the images, especially on the PSIO samples. Also notableis that the images of the two different cameras are identical. The quantification of the photocatalytic activity was done by the analysis of the images by the method discussed in Section 4; the results are in Figure 8 and Figure 9.

To calculate the absorbance of red, green and blue colors of the samples, the reference intensities were measured before applying the dye. The spectra show that the absorbance of different colors decreased differently over time during the experiment. Red and green absorbance shows a decrease in every spectrum, in contrast to blue, which hardly follows any tendency. Comparing the spectra made from the images of the Sony A6000 and the Nikon D3400, it is observable that the difference is minimal. Based on this, the photocatalytic effectiveness of the samples was compared by using the relative absorbance of the red and green colors, averaging the data obtained with the two cameras (Figure 9 and Table 3).

**Figure 10:**
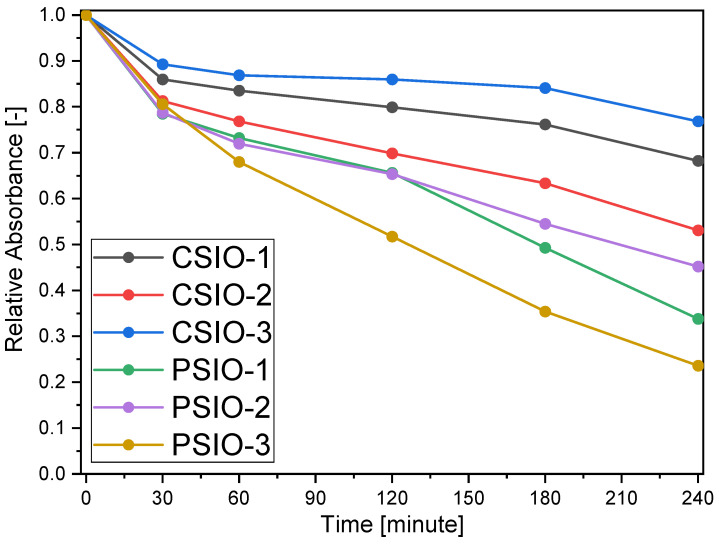
Relative absorbance of the red and green colours of the pixels as a function of time during the photocatalytic test for every sample.

The data from the two cameras were averaged out, and then the red and green curves were added together. These curves (Figure 10) were normalized so the start of the experiment has 1.0 absorbance; thus, a relative absorbance as a function of the time curve can be obtained for each of the samples. Also, using the Lambert–Beer law, an estimate can be made of the proportion of the residual methylene blue.

**Figure 11:**
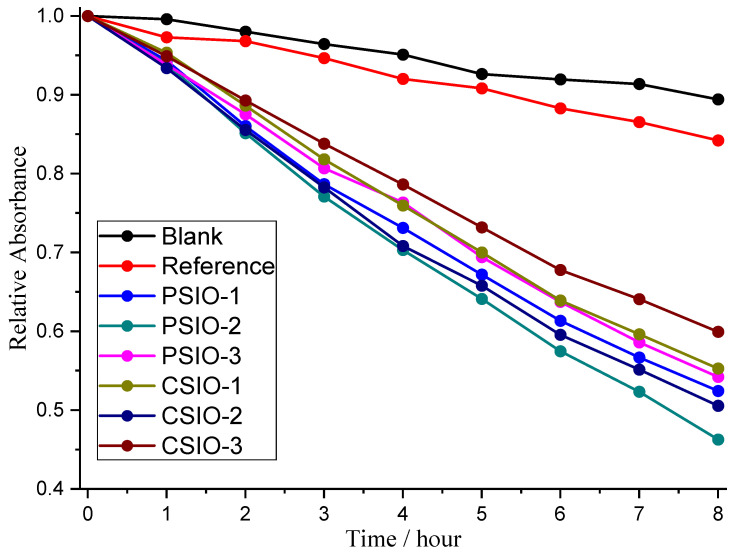
Visible light photocatalysis of rhodamine 6 G by the reference and photocatalysts.

Visible light degradation of rhodamine 6 G was performed using the photocatalysts, using a TiO_2_ layer as a reference (Figure 11). The experiment showed that the dye photodegrades 10.6%, but TiO_2_ is not optimal for visible light catalysis, with degrading of 15.8% of the dye. It is observable that the best-performing inverse opals are the PSIO-2 and CSIO-2, with 53.7% and 49.4%, respectively. This indicates that the ideal wall thickness is close to the layer thickness, which fully fills up the opal structure. The next thickness is the thin layer, which is 47.6% and 44.7% for PSIO-1 and CSIO-1, then the thick layer, which is 45.8% and 40.1% for PSIO-3 and CSIO-3. The mechanism behind the wall thickness can be a combination of the inner surface area of the inverse opal, transparency to the incoming light and the position of the photonic properties. The PSIO samples performed better than their same-wall-thickness counterparts, but not as much as under UV irradiation. The latter can indicate the advantage of disorder in visible light photocatalysis [21].

Photothermal catalysis was tested by temperature measurement during a 2 h period of the photocatalysis (Figure S1 and Table S1). The starting temperatures were at 23.0–25.0 °C the solution heated up to 35.9–37.0 °C. There is little difference between the samples, but, interestingly, the highest temperature was achieved by the PSIO-2 and CSIO-2 inverse opals, then PSIO-2 and CSIO-2, with 36.5 and 36.6 °C and finally the PSIO-3 and CSIO-3, with 36.3 and 36.0 °C. This seems like the same order as visible light photocatalysis; however, it is a small, sometimes sub-1 °C difference. On the other hand, the reference TiO_2_ also heated up the solution to a significant 36.8 °C. TiO_2_ is also a researched photothermal catalyst [45], and it has a temperature-dependent photocatalysis mechanism [46].

## 3. Discussion

TiO_2_ inverse opals were fabricated from the vertical deposition of polystyrene and carbon nanospheres, TiO_2_ layer deposition using ALD and heat treatment to achieve the inverse opal structure. Simple geometric calculations showed what structure we should expect relating to the interstitial sites of the six samples. Structural characterization with SEM and FIB-SEM reveals that the inverse opal structures were successfully prepared; also, the TiO_2_ layer was detected by EDX and the anatase crystalline form was confirmed with XRD. Diffuse reflectance UV-Vis spectra show no photonic properties for the CSIO samples, but for the PSIO samples, they revealed some absorption enhancement. Reflectance mode UV-Vis spectra appear to be more complex; in most cases they showed the photonic band gap, and the “slow” photon caused absorption enhancement. FDTD simulations explain the inverse opals’ optical response. Three regions are distinguishable, where a different mechanism dictates the optical properties: the first region is the absorption edge of the TiO_2_, the second region is highly dependent on the geometry of the inverse opal, and in the third region, the inverse opal starts to act like a single slab of TiO_2_. Simulations also showed that the carbon nanosphere-based inverse opals’ spectra are highly affected by the disorder, merging optical regions two and three. Photocatalytic experiments showed that the samples are capable of degrading methylene blue from the catalyst’s surface. Even though the carbon nanospheres have a much higher deviation of diameter than the polystyrene nanospheres, the inverse opals based on them still show photonic capabilities and photocatalytic activity. Under visible light, the preferred wall thickness is the one closer to fully infiltrating the opal crystal, but no more. However, a thinner layer thickness performs better than a thicker one. CSIOs are closer in efficiency under visible light than under UV, which could indicate that disorder can be beneficial for visible light harvesting, as described in [21]. In conclusion, the use of carbon nanospheres for visible light photocatalysis is less optimal than polystyrene, but the difference in efficiency and price might make it more suitable for large-scale industrial use. Similarly, the partial filling of the opal structure can decrease the inverse opals efficiency but can reduce the costs of the ALD deposition.

## 4. Materials and Methods

**Figure 12:**
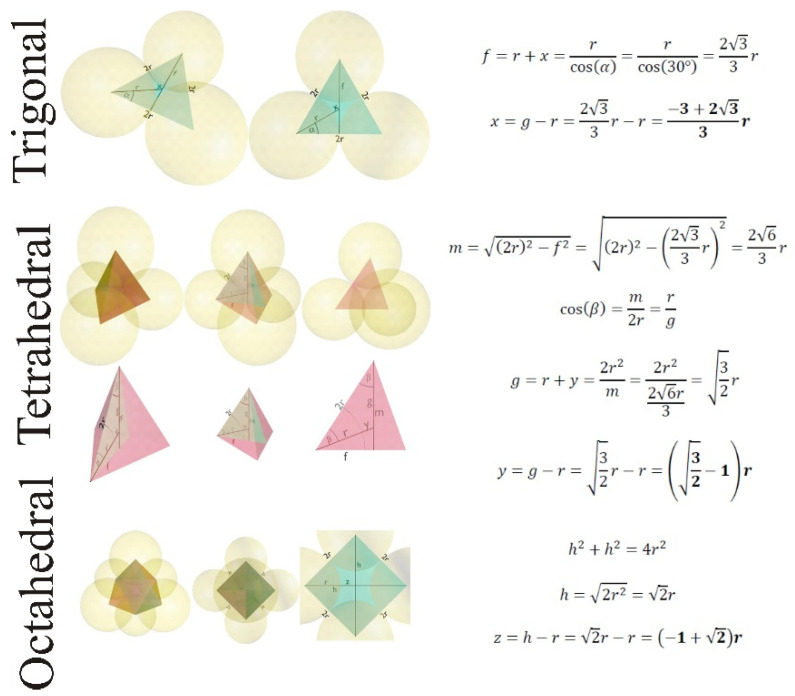
Representation of the interstitial sites and the equations used in the calculation of their size.

Calculations on the inverse opal structure were done to adjust the ALD cycle number, creating these hollow materials with different wall thickness. There are three interstitial sites in a crystal structure: trigonal, tetrahedral and octahedral. To calculate how to fill the template fully and partially, colloid crystal, calculations are needed. The trigonal interstitial site is the smallest one and thus acts as a bottleneck during the infiltration; this means that the samples with the thickest wall are the ones where these interstitial sites are filled, and the rest of the samples are partially filled. Figure 12 shows an image of the interstitial sites made by Blender 2.8 and the equations used in the calculation to estimate the ALD cycle numbers needed and the size of the residual cavities.

In Table 4, we can see the calculated radius of the spheres, that fit inside the interstitial sites of the opal structure. Using the deposited layer thickness completed by ALD, it is possible to calculate all the remaining interstitial sites (tetrahedral and octahedral) inside the opal structure (Table 5). The diameter of the nanospheres is 300 nm for polystyrene and 458 nm for carbon nanospheres, which means that the carbon-templated inverse opals will have larger cavities overall. In conclusion to the calculations, we can see that PSIO-1 is partially coated and has cavities from the remaining trigonal, tetrahedral and octahedral interstitial sites. The trigonal interstitial sites PSIO-2 and -3 are closed; due to this, their other sites have the same size, but the latter have a thicker outer layer on the surface of the sample. The CSIO samples all have different interstitial site sizes, and only CSIO-3 has its trigonal site closed.

The carbon nanospheres with 458 nm average diameter were prepared by the hydrothermal method from sucrose in the form of black powder. The exact preparation method is the same as was described in an earlier article [44]. Polystyrene nanospheres of 300 nm average diameter were purchased from Sigma Aldrich in the form of 10 wt% water suspension.

For the synthesis of the colloid opal crystal, the first step is to make a 0.3 wt% suspension out of the carbon nanosphere powder and the polystyrene suspension; from this point, the preparation of the colloid crystal is the same for the polystyrene and the carbon nanospheres. The nanosphere suspension was placed in an ultrasonic bath for 2 h to achieve the disaggregation of the spheres. Meanwhile, the substrates (microscope glass) were cleaned with soap and ethanol and then treated with piranha solution (3:1 mixture of concentrated sulfuric acid and 30% hydrogen peroxide) for 1 h. The next step is the vertical deposition. For this, the glass slides were put vertically in the suspensions and heat treated in a furnace (Nabertherm L9/11/B410) with the following heat program: 50 °C for 14 h, then 80 °C for 1.5 h.

The TiO_2_ layer depositions were carried out with a Beneq TFS-200-186 flow ALD (Beneq, Espoo, Finland) reactor. The layer deposition was done at 53 °C temperature and 1.345 mbar pressure. The ALD deposition cycle was the following program: 300 ms TiCl_4_ precursor, 3 s N_2_ purge, 300 ms H_2_O precursor and 3 s second N_2_ purge. This cycle program was repeated for 213, 426 and 639 times to achieve 15, 30 and 45 nm TiO_2_ layer thicknesses. Clean glass substrates were put in the ALD reactor chamber during the sample coatings to measure the deposited layer thicknesses. Profilometry was used to check the layer thicknesses made by the aforementioned ALD programs for the 213, 426 and 639 cycles, which were respectively, 16.5, 34.3 and 49.7 nm.

The reference for the visible light photocatalysis test was TiO_2_ deposited on an Si wafer with a size of 1.5 × 1.5 cm. The 36 nm layer was deposited with the same ALD program. The TiO_2_ layer was heat-treated by the same furnace program as the inverse opals during the opal template removal. Additional information on the layer is described in the reference [47].

The removal of the polystyrene and carbon nanospheres from the filled inverse opal structure was carried out in a Nabertherm L9/11/B410 furnace. The heat program was the following: the furnace was heated up to 500 °C temperature within 4 h, then remained at 500 °C for 2 h; with this process, the nanospheres were effectively removed from the inner structure.

The SEM images were taken with a LEO 1540 XB (Zeiss, Oberkochen, Germany) scanning electron microscope, in high vacuum mode and using the secondary electron detector. The samples were held in place with adhesive carbon tapes on top of the copper sample holder. For better image quality of the polystyrene and carbon opals, the samples were sputtered with a Au/Pd layer. EDX spectra were taken with a JEOL JSM-5500LV (JEOL, Tokyo, Japan) scanning electron microscope; for each sample, three measurement points were averaged. This was done in a high vacuum, without conductive coating. The inner structure of the inverse opals was examined using a Thermo Fisher Scientific Scios dual-beam scanning electron microscope (FIB-SEM). XRD measurements of the inverse opal samples were made with a PANanalytical X’Pert Pro MPD X-ray diffractometer using Cu K-α radiation, between the angle range of 5°–65°. The diffuse UV-Vis reflectance spectra were acquired with a JASCO V-750 UV–VIS (JASCO, Tokyo, Japan) spectrophotometer equipped with an integrating sphere. For reference BaSO_4_ was used. The reflectance UV-Vis spectra were also recorded with an Avantes AvaSpec-2048 (Avantes, Lafayette, CO, USA) spectrophotometer equipped with optical cables and white Teflon as reference.

The simulations were performed using the FDTD algorithm implemented in Ansys Lumerical FDTD release 2024 R1.3 software. The creation of the numerical model is divided into a few steps and resembles the fabrication process of the inverse opals structure. First, we assume the spheres are tightly packed in three layers in FCC (111) lattice on the PTFE substrate. The dimensions of the spheres correspond to the ones used in the experiment. Next, we cover their surface with a thin TiO_2_ nanolayer. The optical constants of the nanolayer are the same as reported for TiO_2_ thin films deposited using the ALD system [44]. The thickness of the TiO_2_ nanolayers is similar to the ones measured in the experiment, and in the case of the structure made using polystyrene nanospheres, it is equal to 16 nm, 34 nm, and 49 nm, respectively. In the last step, we removed the sphere template to obtain the inverse opal geometry. In the case of PSIO samples, the elementary cell used in the numerical modeling has a width of a single nanosphere. For CSIO samples, it has a width of 4 average-size nanospheres. The Bloch boundary conditions in the x and y directions ensure the presence of long-distance periodicity. In the z direction, we use PML boundary conditions. The structure is discretized uniformly with a resolution of 1 nm and illuminated using a plane wave under normal incidence. To be able to compare the numerical results with the experimental, the gathered absorbance spectra were post-processed to account for the temporal incoherence of the light source (assumed coherence length 15 microns).

Photocatalytic activity was tested by multiple methods, but the used model dye was the same, namely, methylene blue (66720-100G, CAS 122965-43-9) purchased from Sigma Aldritch (Saint Louis, MO, USA). In every photocatalytic test, 25 µL 1 mM methylene blue solution was dropped on the surface of the inverse opal and left to dry for half an hour. The illumination of the samples with UV light was done by three-three 18 W fluorescent lamps stacked on each other and 5 cm apart from the samples. In the first photocatalytic activity experiment, a JobinYvon Labram Raman spectrometer equipped with an Olympus BX41 microscope and a 532 nm Nd-YAG laser as light source were used to monitor the degradation of the dye. The samples with the dried dye on the surface were measured before and after 19 h of illumination. The spectral ranges of the measurements were between 100–1800 cm^−1^, and two points were measured in each sample. The photocatalytic activity was determined by the most intensive methylene blue peak between 1560 and 1700 cm^−1^. The spectra (Figure 7) were normalized by the most intensive TiO_2_ peak, and then the area of the most intensive methylene blue peak was calculated before and after the 19 h irradiation. In the second experiment the samples were photographed every 30 min with a Sony A6000 camera positioned perpendicularly to the samples and equipped with Sony 16–50 mm f/3.5–5.6 objective in 16 mm focal length, 1/80 s shutter speed, f/5.6 aperture value and 100 ISO sensitivity, and a Nikon D3400 DSLR camera but in a 60° angle to the samples with the Nikon 18–55 mm f/3.5–5.6 AF-P objective 1/30 s shutter speed, f/13 aperture value and 100 ISO sensitivity. The cameras were fixed to maintain their position, and the samples were put into a sample holder, so the positions of the samples in the photos were always the same. Photos of the samples were taken every 30 min during the 4 h long photocatalytic experiment. The RGB color composition of pictures was evaluated using Adobe Photoshop CC 2019 20.0.4 release software to adjust the parts of the images where the sample with the dried methylene blue were located, and the ImageJ photo 1.53c analyzer software was used to extract the color intensities (in the RGB scale) of the pixels in 8-bit scaled between 0–255. The sample without the dried solution was used as reference to calculate the absorption values for the three colors (red, green and blue) and to determine it in every half hour during the 4 h long UV irradiation. The Lambert–Beer law was used to determine the residual methylene blue on the samples surface. The utilization of digital cameras made it possible to monitor the whole surface of the samples (where dye was present) over time compared to the Raman spectroscopy method, where only a specific section of the sample could be analyzed.

Visible light photocatalysis tests were carried out using 1.5 × 10^−5^ M Rhodamin 6 G (Cat. No: 419010250 25GR) model dye purchased from ThermoScientific (Waltham, MA, USA). In our experience, methylene blue photodegrades a lot under visible light irradiation; that is the reason we switched to rhodamine 6 G. The catalyst was attached to the wall of a 30 mL cuvette, and then 10 mL of the rhodamine 6 G solution was added; it was coated with parafilm to prevent evaporation. The cuvettes with the catalyst and solution were put away in the dark for a night to allow the system to reach its adsorption equilibrium. A quantity of 3 mL of the solution was taken from the cuvette and measured using an Avantes AvaSpec-2048 spectrophotometer in transmittance mode, then every 1 h after turning the six 18 W visible light fluorescence lamps on. The six lamps were put into two stacks of three facing each other with 10 cm between them; the cuvettes were put in the middle.

The absorbance spectra of 10^−7^ M methylene blue and 1.5 × 10^−5^ rhodamine 6 G in solution were measured. The methylene blue spectrum was taken using reflectance UV-Visible spectroscopy. The methylene blue was dried on BaSO_4_ with multiple 25 µL droplets to color the BaSO_4_, which was also the reference.

The photothermal catalysis test was done by simulating the first 2 h period of the photocatalysis by measuring the temperature every 10 min of the rhodamine 6 G solution. The solution’s composition and the setup geometry were the same as for the visible light photocatalysis experiment.

## Figures and Tables

**Table 1:** Composition of the inverse opal samples from EDX analysis in m/m%.

SAMPLE	CSIO-1	CSIO-2	CSIO-3	PSIO-1	PSIO-2	PSIO-3
TEMPLATE	Carbon nanosphere	Carbon nanosphere	Carbon nanosphere	Polystyrene nanosphere	Polystyrene nanosphere	Polystyrene nanosphere
DEPOSITED LAYER THICKNESS/NM	16.5	34.3	49.7	16.5	34.3	49.7
O	41.2	38.5	27.0	35.0	33.4	35.9
NA	2.1	1.3	1.2	4.0	2.5	2.6
MG	0.4	0.5	0.2	1.4	0.9	0.4
AL	0.3	0.0	0.9	0.5	0.5	0.4
SI	15.8	11.9	24.4	33.0	25.8	23.9
CA	2.1	1.8	6.4	4.2	3.9	3.2
TI	38.1	45.9	40.0	21.9	33.0	33.4

**Table 2:** Results of the Raman monitored photocatalytic degradation of methylene blue after 19 h UV irradiation.

SAMPLE	CSIO-1	CSIO-2	CSIO-3	PSIO-1	PSIO-2	PSIO-3
RESIDUAL METHYLENE BLUE [%]	0.5	0.0	5.3	4.4	4.2	5.3

**Table 3:** The residual methylene blue on the surface of the sample calculating from the relative absorbance using the red and green pixels.

Sample	CSIO-1	CSIO-2	CSIO-3	PSIO-1	PSIO-2	PSIO-3
Residual methylene blue after 4-h irradiation [%]	68.2	53.1	76.9	33.8	45.2	23.6

**Table 4:** The calculated size of interstitial sites in the opal crystals (distance from the middle of the interstitial site to closest point of a nanosphere).

	Polystyrene (r = 150 nm)	Carbon (r = 229 nm)
Trigonal	23.2 nm	35.4 nm
Tetrahedral	33.7 nm	51.5 nm
Octahedral	62.1 nm	94.9 nm

**Table 5:** Samples with the nanosphere used as template, the ALD layer thickness and the calculated size of residual cavities by interstitial sites (“-”, filled).

Sample	CSIO-1	CSIO-2	CSIO-3	PSIO-1	PSIO-2	PSIO-3
Nanosphere	Carbon	Carbon	Carbon	Polystyrene	Polystyrene	Polystyrene
ALD layer thickness	16.5 nm	34.3 nm	49.7 nm	16.5 nm	34.3 nm	49.7 nm
Trigonal	18.9 nm	1.1 nm	-	6,7 nm	-	-
Tetrahedral	35.0 nm	17.2 nm	16.1 nm	17.2 nm	17.2 nm	17.2 nm
Octahedral	77.9 nm	60.1 nm	59.0 nm	45.6 nm	45.6 nm	45.6 nm

## Data Availability

The raw data supporting the conclusions of this article will be made available by the authors on request.

## Supplementary Materials

The following supporting information can be downloaded at: https://www.mdpi.com/article/10.3390/ijms252312996/s1.
