# Supplementary material for: Photonic Band Gap Engineering by Varying the Inverse Opal Wall Thickness
Source: Int J Mol Sci. 2024 Dec 3;25(23):12996. doi: 10.3390/ijms252312996 (PMC11641796; doi:10.3390/ijms252312996)
Supplement: Supplementary file 1 [file ijms-25-12996-s001.zip › ijms-3287656-supplementary.pdf]

PHOTONIC BAND GAP ENGINEERING BY VARYING THE INVERSE OPAL WALL THICKNESS

Dániel Attila Karajz [1]; Levente Halápi [1]; Tomasz Stefaniuk [2], Bence Parditka [3], Zoltán Erdélyi [3], Klára Hernádi [4], Csaba Cserháti [3] and Imre Miklós Szilágyi [4*]

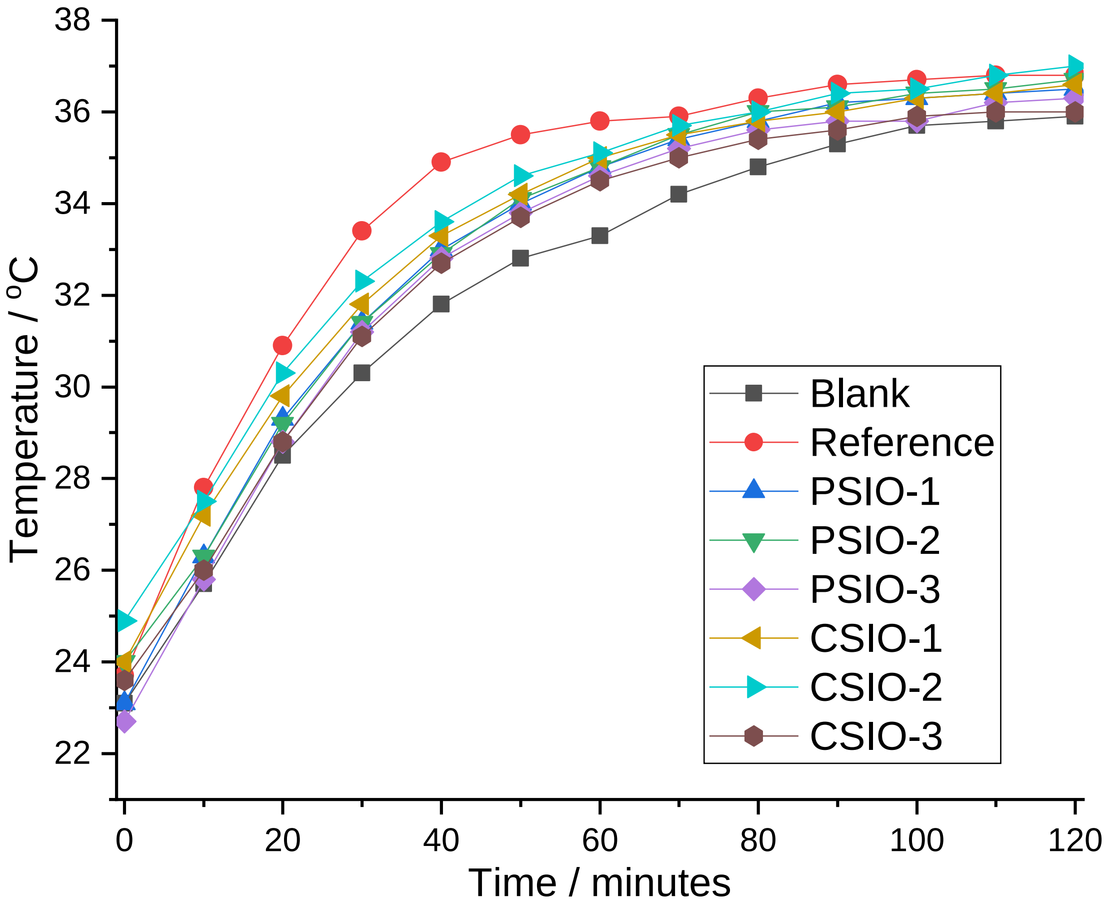

Figure S1. Photothermal catalysis tests

Table S1. Temperature change during the photothermal catalysis test

Time	Blank	Reference	PSIO-1	PSIO-2	PSIO-3	CSIO-1	CSIO-2	CSIO-3
**0**	23.1	23.7	23.1	24.0	22.7	24.0	24.9	23.6
**10**	25.7	27.8	26.3	26.3	25.8	27.2	27.5	26.0
**20**	28.5	30.9	29.3	29.2	28.8	29.8	30.3	28.8
**30**	30.3	33.4	31.4	31.4	31.2	31.8	32.3	31.1
**40**	31.8	34.9	33.0	32.9	32.8	33.3	33.6	32.7
**50**	32.8	35.5	34.0	34.1	33.8	34.2	34.6	33.7
**60**	33.3	35.8	34.8	34.8	34.6	35.0	35.1	34.5
**70**	34.2	35.9	35.4	35.5	35.2	35.5	35.7	35.0
**80**	34.8	36.3	35.8	36.0	35.6	35.8	36.0	35.4
**90**	35.3	36.6	36.2	36.1	35.8	36.0	36.4	35.6
**100**	35.7	36.7	36.3	36.4	35.8	36.3	36.5	35.9
**110**	35.8	36.8	36.4	36.5	36.2	36.4	36.8	36.0
**120**	35.9	36.8	36.5	36.7	36.3	36.6	37.0	36.0

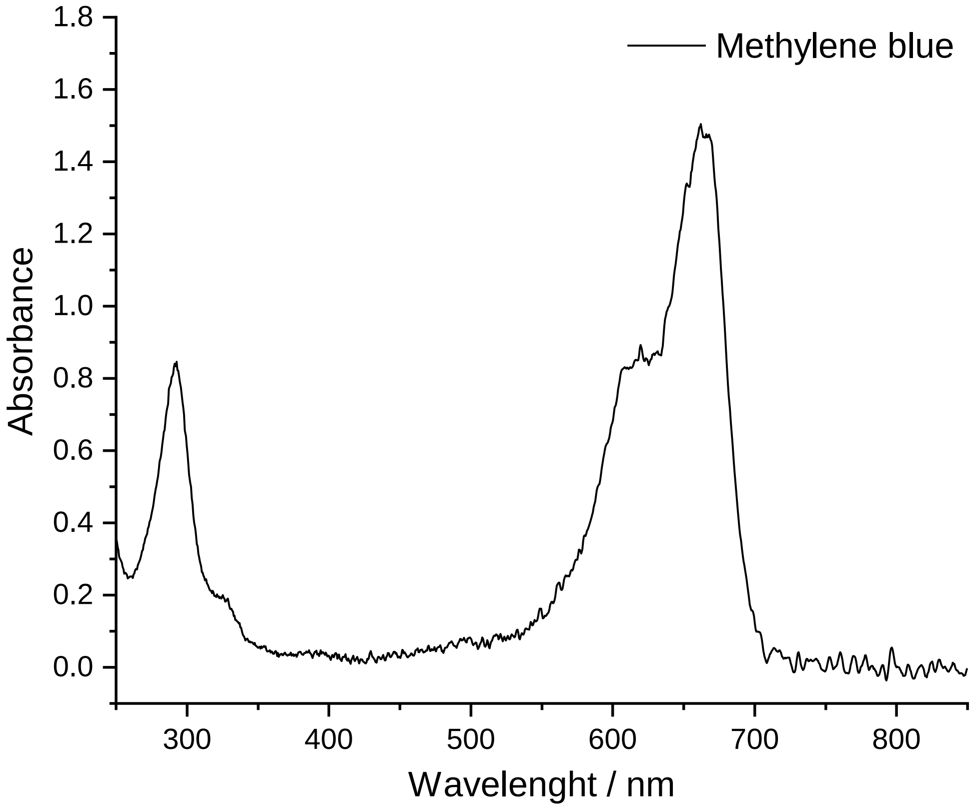

Figure S2. Absorbance spectrum of methylene blue in solution

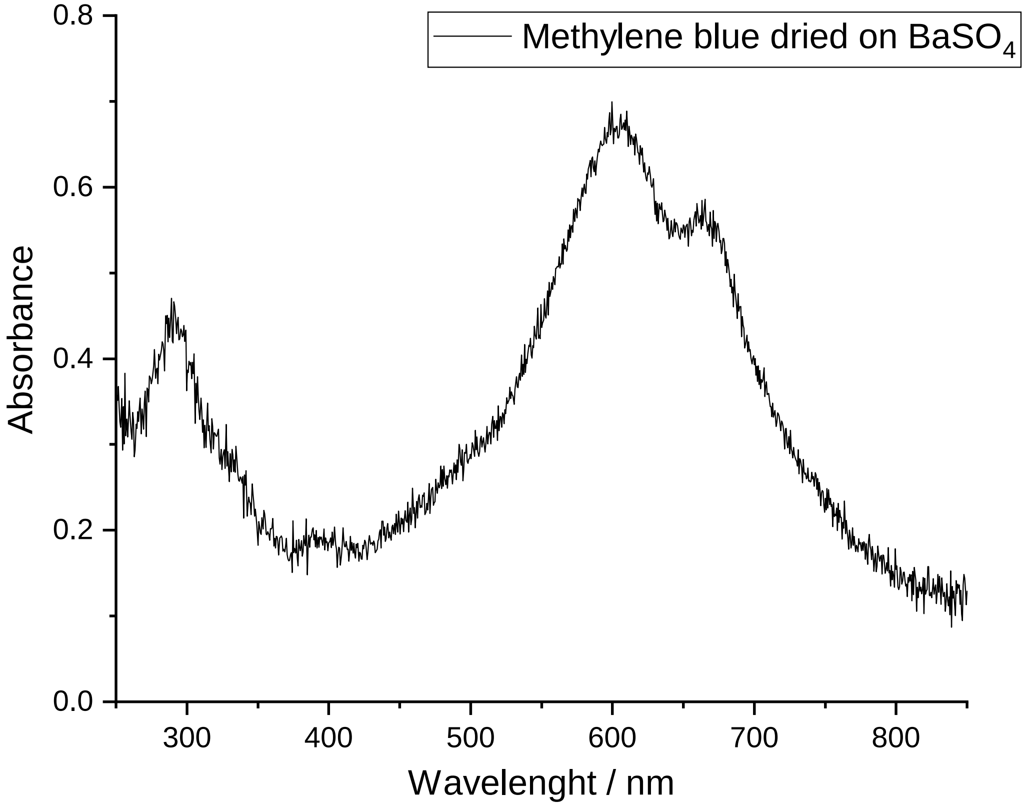

Figure S3. Spectrum of methylene blue on $BaSO_4$

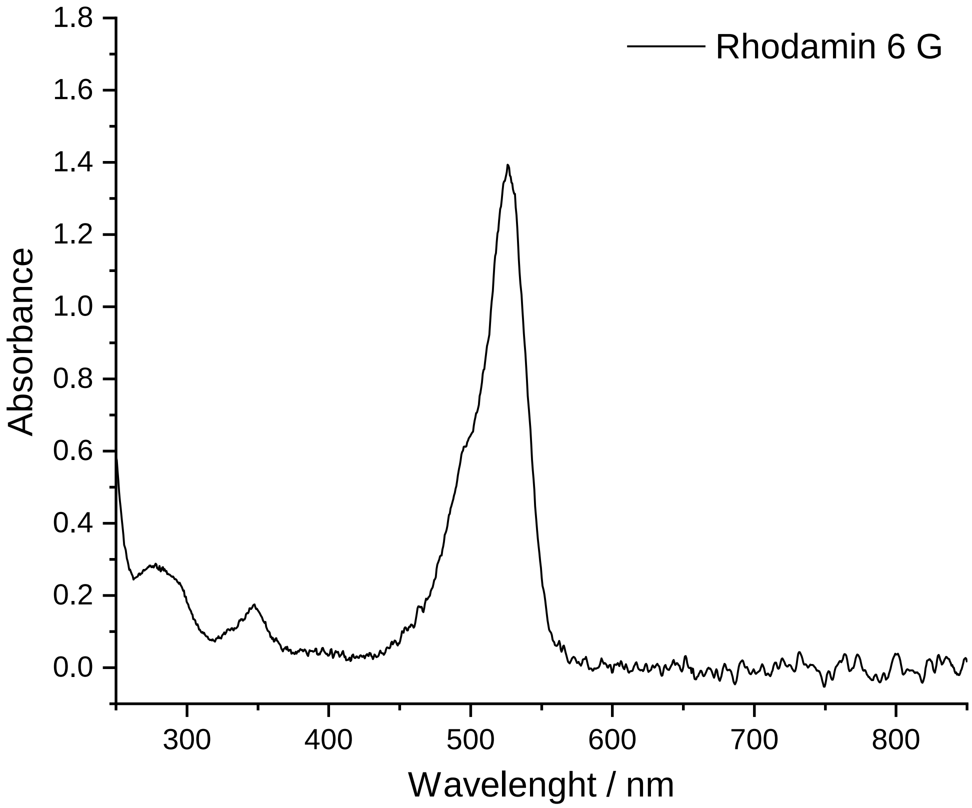

Figure S4. Absorbance spectrum of Rhodamine 6 G in solution
